# Feeling well and doing well. The mediating role of school engagement in the relationship between student well-being and academic achievement

**DOI:** 10.1007/s10212-025-00947-5

**Published:** 2025-03-07

**Authors:** Jakob Schnell, Katja Saxer, Julia Mori, Tina Hascher

**Affiliations:** Department of Research in School and Instruction, University of Bern, Institute of Educational Science, Fabrikstrasse 8, 3012 Bern, Switzerland

**Keywords:** Student well-being, School engagement, Academic achievement, Secondary education

## Abstract

Students’ well-being has become an important part of education policy in many countries. Research shows that well-being contributes to students’ engagement in school, thereby supporting academic achievement. However, prior research has often neglected the interplay and multidimensionality of the constructs. The present study applied a six-dimensional student well-being model and a three-component school engagement model to untangle the differential associations of positive and negative well-being dimensions with the components of school engagement and academic achievement. Longitudinal mediation analyses using a sample of *N* = 754 Swiss secondary school students and two measurement points (Grade 7 and Grade 8) revealed differential associations of well-being dimensions with engagement components, but no direct effects on academic achievement. Enjoyment in school, as a dimension of student well-being, had an indirect effect on academic achievement, mediated through behavioral engagement. The results imply that fostering students’ enjoyment in school may be a promising strategy to enhance their behavioral engagement and, in turn, promote their academic achievement.

## Introduction

In most education systems, school is expected to identify and foster talent, therefore challenging students to achieve to the best of their abilities. To meet the many demands of school, students need to develop a broad set of competencies, while withstanding high time and performance pressure (Hascher et al., 2018). This is especially true within the lower secondary school setting, where substantial academic demands intersect with age-related developmental changes (Virtanen et al., 2019). Coping with these challenges while growing up in an increasingly complex world and facing an uncertain future poses a major risk to students’ well-being. Mental and emotional problems among adolescents in school have increased in recent years, even before the onset of the COVID-19 pandemic (Keyes & Platt, 2023). Such challenges may not only lead to a decrease in student well-being (Widlund et al., 2018) but also in school engagement (Skinner et al., 2008a). Indeed, student well-being and school engagement seem to decline over the school years (Wang & Eccles, 2012), especially after the transition to secondary school (Symonds & Galton, 2014). Recently, awareness of these issues has been raised, and students’ well-being has become an important part of education policy in many countries (OECD, 2019). A rising number of approaches to promote student well-being is being developed and implemented in schools’ curricula, such as positive psychology interventions (Waters, 2011). Fostering student well-being may lead to positive outcomes in multiple ways: First, well-being is an indicator of students’ mental health, both during school years (Antaramian, 2014) and later in life (Carta et al., 2015). Second, it seems likely that promoting well-being can support students’ academic achievement, thereby facilitating the central goal of education (Hagenauer & Hascher, 2010). In other words: To *feel* well in school may help students *do* well in school. However, while students’ well-being and academic achievement appear to be connected, direct links are rarely observed (Bücker et al., 2018). It seems that the influence of well-being on achievement is often more indirect and may be mediated by other variables. One such potential mediator is school engagement (Pietarinen et al., 2014).

Previous research has established relationships between student well-being and academic achievement (Bücker et al., 2018), between student well-being and school engagement (Datu & King, 2018), and between school engagement and academic achievement (Green et al., 2012). Nevertheless, research combining all three constructs is still scarce and clear conceptualizations of these constructs are lacking. The present study aims to shed more light on the potential indirect effects of student well-being on achievement by investigating the mediating role of school engagement. A clearer understanding of how students’ well-being may support their school engagement and how this could be related to their academic success can inform research and practice on creating a positive and supportive learning environment for students. It could offer insights on whether and how interventions aimed at fostering student well-being could also promote school engagement and subsequent achievement. Our longitudinal study in lower secondary education advances research on the multidimensional constructs of student well-being and school engagement and enriches prior literature on the association between these constructs and academic achievement. By distinguishing direct and indirect mechanisms involved in the relationship between student well-being and achievement, it also contributes to differentiating the relative importance of student well-being and school engagement for academic success.

### Student Well-Being

The term *well-being* is currently used abundantly in various contexts, with a plethora of different underlying definitions (Hascher et al., 2018). It is sometimes used as a synonym for happiness, life satisfaction, or the absence of depressive symptoms (Medvedev & Landhuis, 2018). However, such single-dimension conceptualizations of well-being do not capture the complexity of the construct. Diener et al. (2009) coined the term *subjective well-being*, with life satisfaction as a core dimension along with the presence of positive and the absence of negative affect, thereby including cognitive and affective dimensions. This definition makes clear that well-being is considered a multidimensional construct. Still, it does not take the context-specific nature of well-being into account, meaning that a person can experience its dimensions differently in various areas of life. For instance, an adolescent’s general life satisfaction may not necessarily reflect their satisfaction with school (Hascher, 2007). Such context-specific measures need to encompass a variety of aspects related to schoolwork, learning, teachers and peers, to capture student well-being as a whole. In recent decades, a variety of such context-specific definitions of student well-being have been proposed (Hascher et al., 2018). While they often diverge on the specific dimensions, most definitions agree that student well-being is characterized by cognitive, affective, and social aspects related to school (Noble et al., 2008). In our study, we therefore support a multidimensional approach to student well-being, specifically integrating the school context. We align with Hascher (2004) conceptualization of student well-being, defined as the predominance of students’ positive emotions and cognitions toward school, persons in school, and the school context over the negative feelings and cognitions toward school life. This theory-derived concept of student well-being includes six dimensions that cover the broadness of well-being while differentiating relevant dimensions of the school context: enjoyment in school, positive attitudes toward school, positive academic self-concept, and the absence of worries in school, physical complaints in school, and social problems in school. This multidimensional model supports the coexistence of positive and negative factors and incorporates cognitive, emotional, social, and physical factors. It also considers the role of self-esteem as a dimension of subjective well-being (Grob et al., 1991; Veenhoven, 1991).

### School Engagement

Similar to student well-being, school engagement is considered a multidimensional construct and has been defined ambiguously (Upadyaya & Salmela-Aro, 2013). Scholars differ not only in their understanding of which dimensions constitute school engagement, but also on how these dimensions should be conceptualized (Appleton et al., 2008). According to Fredricks et al. (2004), this conceptual unclarity stems from the issue that each type of engagement combines various subconstructs such as interest or effort, which are differently pronounced depending on the research focus. They argue that an engagement measure aimed at predicting broader outcomes like academic success should encompass a multitude of these subconstructs, if only superficially. In consequence, they introduced a conceptualization which divided school engagement into three components—behavioral, cognitive, and emotional engagement (Fredricks et al., 2005). According to this concept, behavioral engagement includes participation in academic and social activities related to school. Cognitive engagement indicates commitment and effort toward school matters, such as the willingness to take the extra steps needed to learn complex school matters and master difficult skills. Emotional engagement refers to affective reactions to teachers, classmates, and school-related events. By integrating behavior, attitude, and affect, this conceptualization of school engagement is considered a “metaconstruct” (Fredricks et al., 2004, p. 60). In our study, we follow this conceptualization, as it allows us to investigate differential effects of engagement components on academic achievement.

### The Relationship between Student Well-being, School Engagement, and Academic Achievement

By definition, student well-being and school engagement seem to share some overlap. Although based on different research domains – well-being research and motivation research –, both constructs include cognitive and emotional components related to school. Some scholars even conceptualize school engagement as part of student well-being. For example, Lan and Moscardino (2019) define student well-being as a combination of academic engagement, satisfaction with social relationships, and commitment to school. However, we argue that student well-being and school engagement are related, but distinct constructs: Student well-being encompasses cognitive and affective *appraisals of* and *reactions to* school and the school life and includes *sources of* well-being (e.g., whether experiences in school contribute to students’ enjoyment). School engagement refers to students’ active *investment in* and *emotional attachment to* school-related activities, with an emphasis on behaviors and intentions that reflect involvement (e.g., whether students actively and enthusiastically approach their schoolwork).

As such, both constructs are associated with academic outcomes. This association is probably reciprocal in nature: Previous studies showed evidence both for the influence of student well-being on school engagement, as for the influence of school engagement on student well-being (Datu & King, 2018). Academic achievement is influenced by school engagement (Green et al., 2012), but has also been shown to affect subsequent school engagement (Salmela-Aro & Upadaya, 2012). Likewise, academic achievement predicts student well-being and vice versa (Morinaj & Hascher, 2022). However, evidence for direct influences of student well-being on academic achievement is often weak and inconsistent (Bücker et al., 2018; Yang et al., 2019). These inconsistencies may be explained by two reasons: First, studies examining this relationship used different and not always school specific operationalizations of student well-being, such as life satisfaction (Z. J. Ng et al., 2015) or general subjective well-being (Steinmayr et al., 2016), instead of multi-dimensional, context-specific measures. Using such unidimensional, general assessments may conflate potential effects of specific student well-being dimensions on achievement, since they could suppress or amplify each other. Second, it can be assumed that the effect of student well-being on achievement is more indirect. Well-being may serve as a facilitator for adaptive school-related attitudes, affect, and behavior, such as school engagement, which could ultimately lead to better learning outcomes (Hascher et al., 2018; Pietarinen et al., 2014). School engagement may therefore function as a mediator between students’ well-being and academic achievement: Student well-being positively predicts school engagement, which in turn may support academic achievement (Gutman & Vorhaus, 2012). While this evidence hints at a causal connection between student well-being, engagement, and achievement, it does not differentiate between specific dimensions of well-being or engagement components. Therefore, it remains unclear which dimensions of well-being may predict which components of engagement. The present study addresses this research gap by integrating six student well-being dimensions and three school engagement components to investigate their differential effects on academic achievement.

Considering the multi-dimensional construct of well-being, it can be expected that different dimensions do not show the same pattern of relationship with engagement. For example, it is reasonable to consider that enjoyment in school is a crucial, but not the sole contributor to emotional engagement, as indicated by research (Ely et al., 2013). Pleasant school experiences may lead to a development of positive emotional involvement with school, such as increased interest in school matters. Positive attitudes toward school may have a primarily positive effect on cognitive engagement, given that positive attitudes, commitment, and effort are related (Fabiny & Lovaš, 2018). Similarly, it can be assumed that the different engagement components are not equally contributing to academic achievement. Previous studies have found relationships between all three engagement components and achievement, with the strongest evidence for behavioral engagement (for a meta-analysis see Lei et al., 2018). Furthermore, it is unclear whether the impact of well-being on academic achievement is mainly mediated by engagement, or if some dimensions of well-being directly contribute to achievement over and above school engagement. This distinction has important implications for both theory and practice. If the association between well-being and achievement is mediated by engagement, then promoting both student well-being and engagement simultaneously may need to be addressed in fostering academic achievement. If there is a relationship between well-being and achievement beyond engagement, it may be essential to put more emphasis on a school environment that cultivates well-being. Also, it must be considered that other factors than engagement may play a role in the relationship between well-being and achievement, such as achievement goal orientations (Holzer et al., 2022).

Two theoretical models provide complementary explanations for the indirect pathway from student well-being to academic achievement through school engagement. According to the broaden-and-build theory (Fredrickson, 2001), the experience of positive emotions leads to an expanded thought-action repertoire and creates urges to take in new information and experiment, thereby facilitating learning, The experience of negative emotions, on the other hand, narrows one’s thought-action repertoire, which hinders learning. Previous studies have used the broaden-and-build theory to explain the relationships between student well-being and school engagement (Datu & King, 2018), and between student well-being and academic achievement (Bücker et al., 2018). By following this theory, we therefore suggest a causal connection: Student well-being shapes the ground for commitment to, interest and participation in school (i.e., school engagement), which boosts academic achievement. In the school context, previous results confirmed that positive experiences can foster engagement and subsequent performance (King et al., 2015).

The broaden-and-build theory focuses mainly on affective processes and does not fully explain the associations between cognitive and social aspects of well-being with engagement and achievement. The self-determination theory (SDT; Ryan & Deci, 2000) provides a more detailed explanation for these associations. According to this theory, all human beings hold basic needs for competence, autonomy, and relatedness. Well-being may be an indicator for satisfaction of these needs (Niemiec et al., 2006). At the same time, basic need satisfaction serves as a resource for motivation and is positively related to students’ engagement (Skinner et al., 2008a) and achievement (Buzzai et al., 2021). To date, no research has linked the six-dimensional well-being model used in our study with basic need satisfaction. Still, SDT can theoretically explain the connection between specific well-being dimensions with engagement components and academic achievement. In the following, we present partial evidence for each of the six well-being dimensions and link it to SDT.

Sufficient need satisfaction may be a positive indicator of all three positive student well-being dimensions (i.e., enjoyment in school, positive attitudes toward school, positive academic self-concept) and predict school engagement and subsequent academic achievement.

It has been shown that when students’ basic psychological needs are met, students are more likely to experience schoolwork as joyful (Shernoff et al., 2003). *Enjoyment* in school, in turn, was found to positively affect achievement, and this effect was mediated through behavioral engagement (Kang & Wu, 2022). Likewise, basic need satisfaction may lead to *positive attitudes* toward school, which are able to reinforce behavioral engagement, thereby fostering academic achievement (Green et al., 2012). Positive self-evaluations of competence are related to students’ *academic self-concept*, which has proven to be an important antecedent of behavioral engagement that in turn predicts academic achievement, even for students with low cognitive and emotional engagement (Schnitzler et al., 2021).

Insufficient need satisfaction, on the other hand, may be related with the negative student well-being dimensions (e.g. worries in school, physical complaints in school, and social problems in school), diminishing school engagement, and subsequent achievement. Pekrun et al. (2002) linked psychological need thwarting to *worries* in school and drew a path from worries to less interest and effort, resulting in lower academic achievement. Interest and effort are often conceptualized as factors of emotional and behavioral engagement (Groccia, 2018). *Social problems* in school may be an indicator of insufficient social relatedness, which in turn can negatively affect school engagement and academic achievement. Students who experience social problems with teachers and peers tend to exhibit less behavioral engagement and lower academic achievement (Olivier et al., 2018). Conversely, Pietarinen et al. (2014) showed that having positive social relations with teachers and peers in school impacts cognitive engagement both directly and mediated through student well-being, and that cognitive engagement positively predicts academic achievement. *Physical complaints* in school can be a symptom of insufficient need satisfaction as well. For example, peer victimization, which gravely harms students’ need for relatedness, has been linked to somatic complaints in previous studies (Nixon et al., 2011). Regarding the effects of physical complaints on school engagement and achievement, Conner and Pope (2014) found negative correlations between physical health symptoms and cognitive and affective engagement, as well as academic achievement.

Taken together, prior results suggest a connection between all dimensions of student well-being with school engagement components, in particular behavioral engagement, and academic achievement. However, the operationalizations of engagement in the presented studies differ, which limits the comparability of results. For example, Kang and Wu (2022) measured behavioral engagement using four items from the Engagement vs. Dissatisfaction with Learning Questionnaire (Skinner et al., 2008b), while Green et al. (2012) conceptualized behavioral engagement as a combination of class participation, homework completion, and absenteeism*.* Also, no previous study included all six well-being dimensions and all three engagement components simultaneously to investigate their associations with achievement. Additionally, when investigating the relationship between student well-being, school engagement, and academic achievement, it may be important to consider possible confounding variables. One potential confounding variable is students’ socioeconomic status (SES), since it is related to both well-being and achievement (Bücker et al., 2018; OECD, 2019). School engagement of students from lower SES families is more likely to diminish over the school years (Y. Li & Lerner, 2011), which may contribute to achievement gaps between these students and their economically more advantaged peers (C. Ng et al., 2018). At the same time, school engagement can serve as a protective factor, as it may moderate the relationship between SES and academic achievement (L. Li et al., 2022). Since lower SES has also been negatively linked to student well-being (Hascher et al., 2018), research on the link between well-being, engagement, and achievement would profit from taking students’ socioeconomic background into account.

### The Present Study

Evidence suggests a connection between student well-being, school engagement, and academic achievement. However, previous studies concerning the relationship between student well-being and school engagement often measured either construct uni-dimensionally or included only certain aspects, neglecting other dimensions (Appleton et al., 2008; Upadyaya & Salmela-Aro, 2013). Additional unclarity comes from the fact that the same scale items have been used to measure different components of engagement across studies (Jimerson et al., 2003). The present study aims at resolving some of the conceptual unclarity regarding the relevant constructs and their associations by applying a multi-dimensional approach.

Both academic achievement and student well-being may be considered as hallmarks of good schooling in the twenty-first century (OECD, 2019). Previous research has shown that well-being and achievement are connected (Bücker et al., 2018), and that this connection can be influenced by school engagement (Pietarinen et al., 2014). Although student well-being, as well as school engagement, have proven to be malleable and can be fostered in school (Abbot, 2017; Waters, 2011), it remains unclear which aspects of the multidimensional constructs are especially important for students’ academic achievement. The present study is among the first that analyses the relationship between six student well-being dimensions and three school engagement components with the same sample, thereby following the suggested multi-dimensional conceptualization of the constructs.

**Fig. 1:**
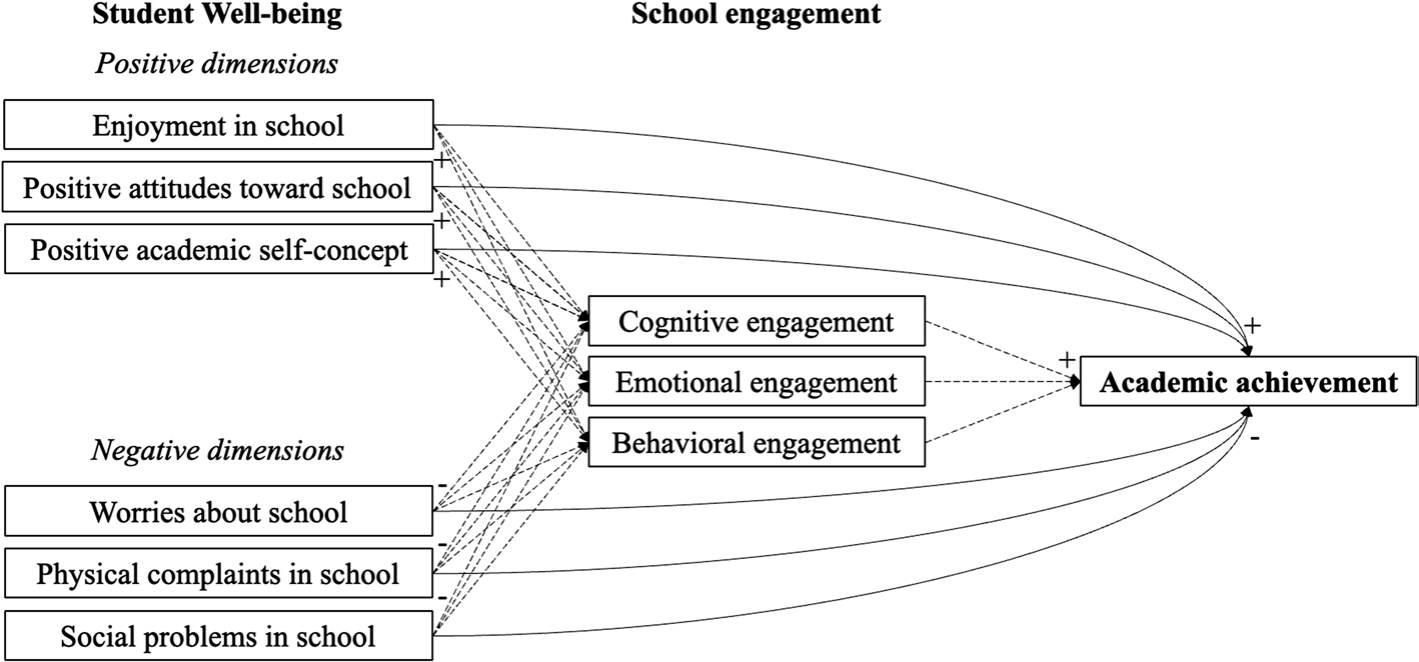
Mediation model for the effects of student well-being on academic achievement through school engagement

The aim of the present study was to examine how dimensions of student well-being are associated with school engagement components and how these associations are related to students’ academic achievement. Specifically, we were interested in the differential effects of positive and negative dimensions of student well-being on the emotional, cognitive, and behavioral components of engagement. Based on previous research, we expected positive relations of the positive student well-being dimensions (Hypothesis 1) and negative relations of the negative student well-being dimensions with achievement (Hypothesis 2). We also expected positive relations of the positive student well-being dimensions (Hypothesis 3) and negative effects of the negative student well-being dimensions with school engagement (Hypothesis 4). Further, we expected all three components of school engagement to positively relate with achievement and mediating the relationship of student well-being with achievement (Hypothesis 5). The proposed mediation model is displayed in Fig. 1.

## Material and methods

### Participants and Procedure

Participants of the study were 754 lower secondary school students from three German-speaking cantons in Switzerland who participated in the longitudinal research project “Well-being in School in Switzerland” (WESIR), funded by the Swiss National Science Foundation (SNSF). Prior to the project start, a study and data management plan were presented to and approved by the SNSF. The study has not been preregistered otherwise. Ethical approval was obtained from the ethics committee at the researchers’ university prior to data collection (Ethics Application Nr 2021-08-00005, August 2021). Written consent for students’ participation in the study was obtained from their parents. Students were informed that their participation was optional and were assured that the information they provided would be confidential. Participants filled out an online survey during regular school lessons with both a teacher and a member of the research team present in the classroom. It took about 90 min to complete the whole survey. The survey was administered at two time points: The first wave of data collection (*t*1) was conducted between January and April 2022, when participants were in Grade 7. The second wave (*t*2) was conducted between January and April 2023, when participants were in Grade 8. 46 classes from 17 schools participated at *t*1, with a total *N* of 754 students (*M*_age_ = 13.12 years, *SD* = 0.59; 48% female). One school dropped out of the study prior to *t*2. Thus, 43 classes participated at *t*2, with a total *N* of 719 students (*M*_age_ = 13.92 years, *SD* = 0.81; 48% female).

### Measures

#### Student Well-being

Student well-being was measured using the 19-item Student Well-being Questionnaire (SWBQ; Hascher, 2007), which contains six subscales: (1) Enjoyment in school (3 items, e.g., “*In the past few weeks, it occurred that I was happy because I could do something I enjoy in school”*), (2) Positive attitudes toward school (3 items, e.g., “*I like going to school”*), (3) Positive academic self-concept (3 items, e.g., “*I have no problems with meeting the school requirements”*), (4) Worries in school (3 items, e.g., “*In the past few weeks, it occurred that I worried about school”*), (5) Physical complaints in school (4 items, e.g., “*In the past few weeks, it occurred that I had strong headaches during class”*), and (6) Social problems in school (3 items, e.g., “*In the past few weeks, it occurred that I had problems with my classmates”*). Responses were indicated on a 6-point Likert scale ranging from 1 = *never/disagree* to 6 = *very often/agree*. The internal reliability of the subscales as indicated by McDonald’s ω ranged from 0.71 to 0.83. All items are presented in Table 4 in Appendix 1.

#### School Engagement

School engagement was assessed using the 19-item School Engagement Scale (Fredricks et al., 2005), comprising three subscales: (1) Behavioral engagement, which measures participation and involvement in academic activities (5 items, e.g., “*I pay attention in class”*), (2) Cognitive engagement, which measures thoughtfulness and effort (8 items, e.g., “*I check my schoolwork for mistakes”*), and (3) Emotional engagement, which measures positive and negative reactions to teachers, classmates, academics, or school (6 items, e.g., “*I feel excited by my work at school”*). Responses were indicated on a 5-point Likert scale ranging from 1 = *never* to 5 = *all of the time*. The internal reliability of the subscales as indicated by McDonald’s ω ranged from 0.73 to 0.84. All items are presented in Table 5 in Appendix 1.

#### Academic Achievement

Academic achievement was measured using grade point average (GPA), which was computed based on students’ grades in mathematics, German (school language), French (first foreign language), English (second foreign language), nature and technology, and history received from teachers at the end of the school years. The school grades varied from 1 (the lowest achievement level) to 6 (the highest achievement level), indicating that a higher score represents a better grade. Since there are no mandatory standardized achievement tests in Swiss schools and grades serve as the common indicator to evaluate students’ school success used by teachers and officials, GPA was deemed the most useful informant of academic achievement.

#### Socioeconomic Status

Socioeconomic status was operationalized as economic, social, and cultural status (ESCS) using the PISA 2018 framework (OECD, 2019). ESCS is an index of highest parental occupation, highest parental education, and various household possessions such as the number of books at home.

### Data Analysis

To explore the relationship between student well-being, school engagement, and academic achievement, mediation analyses using structural equation modeling (SEM) were conducted. Due to the complexity of the model, we performed separate SEMs for each student well-being dimension. Student well-being dimensions and school engagement components were included as latent variables. We tested for direct effects of student well-being at *t*1 on school engagement and academic achievement on *t*2, and for indirect effects of student well-being on academic achievement via school engagement. Statistical significance of the direct and indirect effects was tested using bias-corrected bootstrapping confidence intervals based on 10,000 bootstrap draws at the 0.05 level. In all analyses, we controlled for ESCS and prior academic achievement. The hierarchical data structure of students nested within school classes was controlled for using cluster-robust standard errors. The proportion of missing values on the item level ranged from 13.8% at *t*1 to 18% at *t*2. Because we did not receive grade reports from all participating schools, the missing values for academic achievement ranged between 14.7% at *t*1 and 37% at *t*2. We performed Little’s (1988) test to check whether the missings were completely at random (MCAR). The MCAR test for the survey variables was nonsignificant (χ^2^(38) = 42.73, *p* = 0.275), indicating that the missing data was MCAR. Thus, to deal with the missing values, we employed the full information maximum likelihood approach (FIML).

Due to the conceptual overlap between student well-being dimensions and school engagement components, we conducted various post-hoc analyses to corroborate our results and rule out potential confounding issues. First, we checked intercorrelations on the item level. We assessed whether items from the same subscales correlated relatively high with each other and lower with items from different subscales.

Second, we compared a series of exploratory factor analyses (EFA) to test the validity of the proposed factor structure and to identify potentially problematic cross-loadings for items from the engagement subscales on well-being factors. Items were considered problematic if they had cross-loadings above 0.32 (Tabachnick & Fidell, 2001).

Third, we compared a series of confirmatory factor analyses (CFA) to test the validity and distinctiveness of the student well-being and school engagement constructs. We compared eight models: (1) A one-factor model with all student well-being and school engagement variables loading on a single factor; (2) a two-factor model with one higher-order factor for all well-being dimensions and one higher-order factor for all engagement dimensions; (3) a two-factor model with one higher-order factor for the positive well-being dimensions together with the engagement components and one higher-order factor for the negative well-being dimensions; (4) a three-factor model with one higher-order factor for the positive student well-being dimensions and emotional engagement, one higher-order factor for the negative student well-being dimensions and one higher-order factor for the other two school engagement components; (5) a three-factor model with one higher-order factor for the positive student well-being dimensions, one higher-order factor for the negative student well-being dimensions and one higher-order factor for the school engagement components; (6) an eight-factor model with positive attitudes toward school and emotional engagement as one factor and all other dimensions as separate factors; (7) an eight-factor model with enjoyment in school and emotional engagement as one factor and all other dimensions as separate factors; (8) a nine-factor model with each well-being dimension and each engagement component loading on a separate factor. Given the conceptual similarity between enjoyment in school, positive attitudes toward school, and emotional engagement, models 4, 6, and 7 were specified with combinations of those dimensions to test their distinctiveness.

Data preparation, descriptive and correlation statistics were conducted using R version 4.0.3 (R Core Team, 2020). CFA, EFA, and SEM analyses were performed in MPlus 8.10 (Muthén & Muthén, 1998–2017). Results were imported back to R using the mplusautomation package (Hallquist & Wiley, 2018). This package allows to convert the MPlus output to an R data frame, which facilitates the extraction and organization of results.

## Results

### Descriptive statistics

Means, standard deviations, reliability coefficients, and bivariate correlations are presented in Table 1. As expected, the positive student well-being dimensions positively correlated with each other, as did the negative dimensions, and positive and negative dimensions correlated negatively. Correlations were low to moderate, except for the high correlations between positive attitudes toward school and enjoyment in school. School engagement components also positively correlated with each other and with the positive well-being dimensions, while correlations with the negative well-being dimensions were negative for behavioral and emotional engagement. Cognitive engagement was positively correlated with worries in school and physical complaints in school. All correlations were low to moderate and significant, except for the associations between cognitive engagement and social problems in school. ESCS had low, but significant positive correlations with positive academic self-concept and emotional engagement, and negatively correlated with worries in school and physical complaints in school. GPA at both time points was highly positively correlated with each other, moderately positive with all three positive well-being dimensions, behavioral and emotional engagement and ESCS, and lowly negatively correlated with worries in school and physical complaints in school.

**Table 1:** Means, standard deviations, reliabilities, and correlations for dimensions of student well-being, school engagement and students’ GPA

Variable	*M*	*SD*	ω	1	2	3	4	5	6	7	8	9	10	11
1. PAS_t1_	4.28	1.04	0.79											
2. EIS_t1_	4.33	1.01	0.71	0.61***										
3. PASC_t1_	4.31	1.02	0.77	0.34***	0.25***									
4. WIS_t1_	3.29	1.42	0.81	−0.14***	−0.09*	−0.33***								
5. PCS_t1_	2.15	1.25	0.83	−0.15***	−0.09**	−0.26***	0.53***							
6. SPS_t1_	1.67	0.97	0.79	−0.21***	−0.13**	−0.14***	0.27***	0.38***						
7. ENGB_t2_	4.00	0.59	0.73	0.30***	0.26***	0.28***	−0.10*	−0.16***	−0.15***					
8. ENGC_t2_	2.63	0.75	0.80	0.27***	0.26***	0.16***	0.10*	0.13**	0.05	0.36***				
9. ENGE_t2_	3.23	0.74	0.84	0.48***	0.40***	0.25***	−0.13**	−0.19***	−0.20***	0.52***	0.45***			
10. GPA_t1_	4.71	0.44	-	0.20***	0.18***	0.40***	−0.19***	−0.20***	−0.07	0.23***	0.08	0.18***		
11. GPA_t2_	4.79	0.46	-	0.19***	0.15**	0.33***	−0.11*	−0.18***	−0.11*	0.29***	0.14***	0.21***	0.80***	
12. ESCS	0.00	0.76	-	0.01	0.03	0.17***	−0.15***	−0.20***	−0.05	0.05	0.04	0.09*	0.25***	0.30***

PAS = positive attitudes toward school; EIS = enjoyment in school; PASC = positive academic self-concept; WIS = worries in school; PCS = physical complaints in school; SPS = social problems in school; ENGB = behavioral engagement; ENGC = cognitive engagement; ENGE = emotional engagement; GPA = Grade Point Average; ESCS = Socioeconomic Status; *M* = mean; *SD* = standard deviation; ω = McDonald’s Omega; *t*1 = Wave 1; *t*2 = Wave 2. *p < 0.05, **p < 0.01, ***p < 0.001

### Path analyses

Table 2 displays all significant direct and indirect effects. The results revealed that no student well-being dimension had a significant direct effect on academic achievement. All positive student well-being dimensions had significant positive direct effects on all engagement components. For the negative well-being dimensions, social problems in school had a negative direct effect on behavioral and emotional engagement, while physical complaints had a positive direct effect on cognitive engagement. Behavioral engagement had a positive direct effect on academic achievement. One significant indirect pathway was found: Enjoyment in school had a positive effect on academic achievement, mediated through behavioral engagement. The full model results including effects of control variables can be found in Appendix 1.

**Table 2:** Significant direct and indirect paths from Student Well-being dimensions to School Engagement components and Academic Achievement

Path	Estimate	*SE*	95% CI
*Direct Effects*			
EIS_t1_ → ENGB_t2_	0.613***	0.092	[0.432, 0.787]
PAS_t1_ → ENGB_t2_	0.516***	0.076	[0.364, 0.661]
PASC_t1_ → ENGB_t2_	0.553**	0.192	[0.371, 1.065]
SPS_t1_ → ENGB_t2_	−0.201**	0.065	[−0.33, −0.075]
EIS_t1_ → ENGC_t2_	0.600***	0.075	[0.432, 0.723]
PAS_t1_ → ENGC_t2_	0.489***	0.060	[0.37, 0.604]
PASC_t1_ → ENGC_t2_	0.438*	0.180	[0.26, 0.937]
PCS_t1_ → ENGC_t2_	0.164*	0.064	[0.03, 0.281]
EIS_t1_ → ENGE_t2_	0.764***	0.090	[0.601, 0.943]
PAS_t1_ → ENGE_t2_	0.713***	0.055	[0.61, 0.821]
PASC_t1_ → ENGE_t2_	0.523*	0.205	[0.337, 1.098]
SPS_t1_ → ENGE_t2_	−0.237***	0.067	[−0.37, −0.111]
ENGB_t2_ → GPA_t2_	0.129*	0.054	[0.027, 0.242]
*Indirect Effects*			
EIS_t1_ → ENGB_t2_ → GPA_t2_	0.079*	0.037	[0.020, 0.173]

EIS = enjoyment in school; PAS = positive attitudes toward school; PASC = positive academic self-concept; WIS = worries in school; SPS = social problems in school; PCS = physical complaints in school; ENGB = behavioral engagement; ENGC = cognitive engagement; ENGE = emotional engagement; GPA = Grade Point Average; ESCS = Socioeconomic Status; 95% CI = 95% bias-corrected bootstrap confidence interval; *t*1 = Wave 1; *t*2 = Wave 2. *p < 0.05, **p < 0.01, ***p < 0.001

### Post-hoc Analyses

#### Inter-item correlations

Inter-item correlations were generally higher between items from the same subscales than between items from different subscales. Some items from the emotional engagement subscale also correlated moderately with some items from the enjoyment in school and positive attitudes toward school subscales. Since these correlations were lower than the intercorrelations within the specific scales, inter-item correlations were ruled out as possible confounding factor in our path analyses. The full item correlation matrix is available in the online supplementary.

#### Exploratory factor analyses

The fit indices of the EFA models improved with the number of factors. Although the 10-factor solution yielded a better fit than the 9-factor solution, the additional factor had an eigenvalue below 1. Therefore, we kept the 9-factor solution to investigate cross-loadings between student well-being and school engagement variables. No item showed cross-loadings above 0.32 on other factors, indicating no confounding effects in our path analyses. The EFA results for all tested solutions are available in the online supplementary.

#### Confirmatory Factor Analysis

The CFA results are presented in Table 3. Each subsequent model yielded better fit statistics than the previous (lower χ^2^ / *df* ratio, higher comparative fit and tucker-lewis indexes, lower root mean squared error of approximation, lower standardized root mean square residual), indicating that student well-being and school engagement are distinct constructs. The fact that the nine-factor model had a better fit than the three-factor model is an indicator that the student well-being dimensions and the school engagement components should be measured as single factors. Further, the models where emotional engagement was specified to load on a common factor with either positive attitudes toward school, enjoyment in school, or both yielded worse fit than the nine-factor model. This underlines the assumption that the well-being dimensions and emotional engagement are distinct constructs. Both the six-factor structure for student well-being (Hascher & Hagenauer, 2020) and the three-factor structure for school engagement (Ramos-Díaz et al., 2016) have been validated in previous studies. The results from the present CFA reflect these findings, supporting the treatment of all student well-being dimensions and school engagement components as separate factors in the path analyses.

**Table 3:** Model fit statistics of the CFAs testing competing models in terms of the factor structure of Student Well-being and School engagement

CFA Model	χ^2^ (*df*)	CFI	TLI	RMSEA	90% CI	SRMR
one-factor model	5837.913 (665)	0.417	0.384	0.094	[0.092, 0.097]	0.126
two-factor model 1	1888.303 (665)	0.861	0.851	0.046	[0.044, 0.049]	0.088
two-factor model 2	1749.556 (665)	0.877	0.868	0.044	[0.041, 0.046]	0.079
three-factor model 1	1717.729 (653)	0.880	0.871	0.043	[0.041, 0.046]	0.075
three-factor model 2	1629.851 (653)	0.890	0.882	0.041	[0.039, 0.044]	0.072
eight-factor model 1	1910.738 (637)	0.857	0.842	0.048	[0.045, 0.050]	0.063
eight-factor model 2	1804.139 (637)	0.869	0.855	0.046	[0.043, 0.048]	0.062
nine-factor model	1478.686 (629)	0.904	0.893	0.039	[0.037, 0.042]	0.056

χ2 = Chi-Square; df = degrees of freedom; CFI = comparative fit index; TLI = Tucker-Lewis Index; RMSEA = root mean squared error of approximation; 90% CI = 90% confidence interval for the RMSEA; SRMR = standardized root mean square residual

Two-factor model 1: Student Well-being, School engagement; Two-factor model 2: Positive Student Well-being dimensions + School engagement, Negative Student Well-being dimensions; Three-factor model 1: Positive Student Well-being dimensions + Emotional engagement, Negative Student Well-being dimensions, Behavioral engagement + Cognitive engagement; Three-factor model 2: Positive Student Well-being dimensions, Negative Student Well-being dimensions, School engagement; Eight-factor model 1: Positive attitudes toward school + Emotional engagement, other dimensions; Eight-factor model 2: Enjoyment in school + Emotional engagement, other dimensions

## Discussion

In the present study, we investigated the relationship between adolescent students’ well-being, their school engagement, and academic achievement in a longitudinal study with two measurement points in Grade 7 and Grade 8. We expected the three positive student well-being dimensions (positive attitudes toward school, enjoyment in school, positive academic self-concept) to positively predict and the three negative student well-being dimensions (worries in school, social problems in school, physical complaints in school) to negatively predict school engagement and academic achievement. Further, we expected all three components of school engagement (behavioral, cognitive, emotional) to positively predict academic achievement, thereby mediating the effect of student well-being.

We found no student well-being dimension to be directly related to academic achievement. Therefore, we have to reject Hypotheses 1 and 2. While we expected direct relations based on our *theoretical* assumptions, our results are comparable to other empirical findings: Yang et al. (2019) found no direct effects of school well-being on academic achievement. They explain their null findings with the assumption that annual grades are rather stable and that well-being might be more dynamically related to daily academic performance. The high correlation between t1 and t2 GPA found in our study corroborates the assumption of annual grade stability. Thus, our measurement of academic achievement may not capture the dynamic interplay between students’ well-being and possible short-term fluctuations in their school performance. Another reason might be the potential influence of third variables not accounted for in our study. For example, the influence of emotions such as enjoyment in school on academic achievement has been shown to be dependent on the interplay with other factors, like motivation or self-regulated learning (Mega et al., 2014). The same holds true for positive attitudes toward school: Although various studies point out a direct link between students’ attitudes and achievement (for a meta-analysis see Petscher, 2010), this link seems to be dependent on students’ motivation such as academic goals and intentions (Abu-Hilal, 2000). Also, while positive academic self-concept is considered to be reciprocally related to academic achievement (Marsh & Martin, 2011), the influence of previous achievement on academic self-concept might be stronger than vice versa. Since we included previous achievement in our analyses, this could explain why we found no direct associations. Additionally, we measured both well-being and academic achievement on a general level and did not investigate subject-specific differences. Some effects of well-being dimensions might only emerge for achievement in certain subjects. For example, it has been shown that worries are a source of task-irrelevant thoughts that block cognitive resources, thereby impairing performance (Keogh et al., 2004). A student who has difficulties with maths might have more of such worries during maths exams than during language exams. Such potential subject-specific relations are not reflected in a general GPA measure as used in our study.

As expected, all positive well-being dimensions showed positive direct relations with all engagement components, leading to acceptance of Hypothesis 3. Hypothesis 4, however, is only partially accepted: Social problems in school was a negative direct predictor of behavioral and emotional engagement, while physical complaints were unexpectedly positively related to cognitive engagement. One plausible explanation for this finding might be that students who have previously experienced school-related physical complaints may increase their engagement in cognitive tasks as a compensatory mechanism. Alternatively, a third variable such as performance pressure could influence both physical complaints and cognitive engagement. This notion is supported by research indicating that performance pressure can exacerbate physical symptoms (Murberg & Bru, 2004) and boost some forms of cognitive engagement, although in an unfavorable way. Greene (2015) differentiates between two forms of cognitive engagement* –* deep and shallow – and ties these forms to different achievement goal orientations. Within this distinction, deep cognitive engagement is induced by mastery goal orientation, i.e., a focus on learning how to master a task, while shallow cognitive engagement is induced by performance goal orientation, i.e., a students’ comparison of their own performance with that of their peers (Pintrich, 2000). Deep cognitive engagement is characterized by adaptive self-regulated learning strategies, such as combining and comparing different pieces of information. In contrast, shallow cognitive engagement encompasses superficial and ineffective learning strategies, such as memorizing answers for tests. Performance pressure could thus induce a performance-oriented learning climate, leading students to shallow cognitive engagement. Since the scale to measure cognitive engagement used in the present study does not differentiate between deep and shallow engagement, this interpretation remains to be tested.

Hypothesis 5 must be largely rejected, as we found only one significant indirect effect: Enjoyment in school indirectly predicted academic achievement, mediated through behavioral engagement. Behavioral engagement turned out to be the sole predictor of achievement in our model. This finding supports the self-determination theory: Enjoyment in school can be seen as a construct that captures students’ basic need satisfaction (Ryan & Deci, 2000), which may lead to higher behavioral engagement and subsequent academic achievement (Green et al., 2012). Additionally, the link from enjoyment in school to academic achievement through behavioral engagement is in line with the broaden-and-build model, which states that positive emotions lead to adaptive learning behavior and thereby enhance academic performance (Fredrickson, 2001).

For the cognitive engagement component, we found no effect on academic achievement. Although many studies point to a positive connection between cognitive engagement and achievement (Lei et al., 2018), Greene (2015) posits that this might depend on the depth of engagement and the corresponding strategy use. As outlined above, cognitive engagement can be differentiated between deep and shallow forms. Shallow cognitive engagement has been shown to negatively predict academic achievement (Greene, 2015). The insignificant effect of cognitive engagement on achievement found in the present study might indicate that students who rely on shallow learning strategies still experience themselves to be cognitively engaged, but that this form of engagement does not translate to academic achievement.

While student well-being and emotional engagement seem to be closely related, as the direct effects of the well-being dimensions on emotional engagement in the regression analysis suggest, they did also not translate to achievement. The nonsignificant result of emotional engagement found in our study is in line with Fredricks (2004), who reported only weak evidence for a direct effect of emotional engagement on achievement. One possible explanation comes from Wang and Degol (2014), who suppose that emotional engagement could serve as a prerequisite for behavioral and cognitive engagement. According to this explanation, high emotional engagement leads to more participation in the classroom and better self-regulation of learning. This explanation is also supported by empirical evidence: Li and Lerner (2013) found students’ emotional engagement to predict later behavioral and cognitive engagement, and a study by Wu and Wu (2020) found a serial link from emotional to behavioral to cognitive engagement, leading to increased academic performance. In other words, having positive emotions and attitudes toward school seems not enough to succeed academically. Rather, when these emotions and attitudes are accompanied by adaptive learning behavior, they are related to students’ achievement.

Besides the relationship between the internal well-being dimensions with engagement and achievement, external factors seem to play a role too, as suggested by the positive effects of socioeconomic status on achievement found in the present study. Socioeconomic status was a significant predictor of academic achievement in four models, but unrelated to school engagement (see Appendix 2 Tables 6, 7, 8, 9, 10 and 11). This finding implies that students from more advantaged socioeconomic backgrounds tend to have better grades, independent from the school engagement they report. This is in line with numerous other studies that proved a connection between socioeconomic status and academic achievement (for a meta-analysis see Sirin, 2005), especially in stratified education systems such as in Switzerland (Hanushek & Woessmann, 2006). Students from more advantaged socioeconomic backgrounds often have more resources at home, such as a quiet learning environment and parents who can support them academically (Thomson, 2018). On the other hand, it is also possible that teachers’ grading behavior is somewhat biased and favors such students (Doyle et al., 2023). Swiss students get tracked into different performance levels between Grade 6 and 7, where the effects of socioeconomic background are strong (Neuenschwander & Malti, 2009). The participants in the present study were in the seventh grade and thus already assigned to different tracks. The finding that socioeconomic status is still associated with academic achievement within these more homogenous groups suggests that students from more advantaged socioeconomic backgrounds tend to have better grades regardless of their academic track.

### Implications for practice

Some recommendations for school practice can be derived from our findings. One key implication is that fostering students’ academic well-being may lead to positive outcomes in their school engagement. Second, when focusing on students’ engagement, it seems most promising to work on the behavioral component. Giving students opportunities and encouraging them to participate in learning activities may be the best way to help them reach their full potential. Teachers can contribute to their students’ behavioral engagement also by giving goal-relevant feedback, as well as by emphasizing mastery-oriented achievement goals and not comparing individual students’ achievements to those of others (Putwain et al., 2018). A third implication pertains to our finding enjoyment in school indirectly predicts achievement through behavioral engagement. This finding underscores the importance of creating enjoyable and engaging learning experiences to promote academic outcomes and therefore backs our premise that feeling well in school promotes doing well in school to some extent. However, we neither found strong links between well-being and academic achievement, nor between school engagement and academic achievement. It seems that other factors play a more crucial role in secondary school students’ academic success.

### Strengths and limitations

The present study has multiple strengths. First, the integration of multidimensional constructs of student well-being and school engagement allows for a more nuanced approach toward understanding the associations between the variables compared to previous studies that used only certain dimensions and components.

Further, this study design clearly distinguishes between student well-being dimensions and engagement components and therefore allows for the examination of the unique contribution of engagement components, in contrast to previous studies that combined different types of engagement in one measure (Fredricks, 2004). The results of our study confirmed that a more differentiated approach to the constructs under investigation is necessary to understand the complex associations between well-being dimensions, engagement components, and achievement. By examining well-being through various dimensions, researchers can better grasp the intricate interplay of these elements in shaping students’ overall sense of well-being. This holistic perspective not only offers valuable insights into the factors that contribute to or hinder engagement and achievement. Moreover, the multidimensional approach is equally crucial for engagement research, as it allows for a nuanced examination of the factors that drive students to engage in school. In essence, adopting multidimensional models not only enhances our understanding of well-being and engagement, but offers important insights on how well-being, engagement and achievement can be fostered simultaneously.

Also, some limitations must be noted. First, student well-being and school engagement were measured through self-reports of students. Since student well-being is a subjective evaluation of one’s cognitions and emotions toward school, it is best measured using self-reports. Still, research using additional measures, such as teacher perceptions of student engagement and observations of classroom behavior, could contribute to additional clarification on the relationships among the variables.

The second limitation is given by the fact that only variables on students’ individual level were investigated. Further studies could add school- and classroom-related variables, such as the influence of school policies or teacher behavior on students’ well-being and engagement.

A third limitation lies in the theoretical and methodological ambiguity of the engagement construct. As Sinatra et al. (2015) pointed out, all three engagement components possibly intersect, and it is likely that measurement of one dimension reflects the other dimensions as well. In addition, the well-being dimension “enjoyment in school” and the emotional component of the engagement construct significantly overlap. This issue must be kept in mind when interpreting the present results and comparing them to other studies that used different conceptualizations of the construct. Future studies could tackle this ambiguity by using alternative, more nuanced instruments to measure school engagement (Reeve et al., 2020).

Fourth, while the data for student well-being and school engagement were collected one year apart, the engagement data and the school grades stem from the same school year. The mediation analysis would have been more straightforward if the time interval between the mediator and the outcome were identical with intervals between the predictors and the mediator. While the student survey on well-being and engagement took place in the middle of the school year, academic achievement was measured using official school records of grades at the end of the school year. Therefore, a time lag between the measurement of the mediator and the outcome was existent, which allows to investigate the influence of perceived well-being and engagement on academic success.

### Directions for future research

Our results indicate that enhancing students’ well-being may lead to increased school engagement, which, in case of behavioral engagement, may enhance academic achievement. At the same time, physical complaints in school were positively related to cognitive engagement in our study. Future research should aim at resolving this paradoxical finding. We see a promising approach to this in the inclusion of learning strategy-related variables, and by differentiating cognitive engagement further into deep and shallow engagement strategies. Examination of the causal relation between achievement goal orientation, engagement, and strategy use could offer valuable insights into the mechanisms involved in the interplay between student well-being, engagement, and achievement (Upadyaya & Salmela-Aro, 2013). Also, possible sequential effects of engagement components should be investigated. While our data suggests that student well-being has strong effects on cognitive and emotional engagement, these engagement components did not lead to higher academic achievement. As Wang and Degol (2014) suggested, this might be due to the serial mediation of emotional through cognitive and behavioral engagement. To explore the relationship between student well-being and academic achievement in more detail, it may be beneficial to use more nuanced measures, such as subject-specific and short-time indicators. While we found no direct associations between trait well-being and overall annual GPA, studies applying multiple measurement points for well-being and academic performance in different subjects might be able to reveal more dynamic mechanisms in these associations. Furthermore, future research could control for students’ characteristics that may impact the association between students’ well-being, engagement, and academic achievement such as gender identity or personality traits.

To further clarify and refine the conceptual relationship between well-being and engagement with regard to their manifold definitions and conceptualizations, it would be helpful to include multiple measures of both constructs within a single study. This approach would enable inferences about the convergent and discriminant validity of each measure, providing insights into where different conceptualizations of well-being and engagement overlap, where they diverge, and whether they genuinely capture two distinct constructs.

## Conclusions

The results highlight the importance of considering the multidimensional nature of student well-being and school engagement. Behavioral engagement was the sole predictor of achievement in our study, and it mediated the indirect effect of enjoyment in school. This result implies that it is not enough to foster students’ enjoyment in school, but that students also need guidance on how to turn their positive emotions into concrete action strategies to be able to succeed in school. While competition and pressure in school might lead students to cognitively engage with school, it might not necessarily foster the use of effective learning strategies. The value of these strategies for learning and achievement should be communicated by teachers and school staff, and room for learning the use of such strategies should be given at schools. Additionally, we found that the negative well-being dimensions had negative effects on school engagement. We therefore emphasize the need to take students’ school-related worries, physical complaints, and social problems seriously, as they seem to be an indicator of low school-related well-being that is detrimental to their school engagement. Thus, teachers and school staff should strive for a school in which students feel well, so they can adaptively engage in their schoolwork and reach their full potential.

## Data Availability

The data that support the findings of this study are not publicly available, because they are part of an ongoing project. MPlus output files, R script, and prior versions of the article manuscript can be accessed under the following link: https://osf.io/3zmsb/.

## Appendix 1

**Table 4:** Student Well-being Scale

*Enjoyment In School (EIS)*
In the past few weeks, it occurred that I was happy because I could do something I enjoy in school
In the past few weeks, it occurred that I was happy because I could show what I have learned
In the past few weeks, it occurred that I was happy because a teacher was friendly and understanding to me
*Positive Attitudes Toward School (PAS)*
I like to go to school
School makes sense to me
Whatever will happen, school is a good thing
*Positive Academic Self-Concept (PASC)*
I don't have problems with meeting the school requirements
I can solve learning problems easily
I'm able to achieve as good as most of my classmates
*Worries In School (WIS)*
In the past few weeks, it occurred that I worried about school
In the past few weeks, it occurred that I worried about the next school years / about the time after school
In the past few weeks, it occurred that I worried about my grades
*Physical Complaints In School (PCS)*
In the past few weeks, it occurred that I lacked appetite because of achievement-stress in school
In the past few weeks, it occurred that I felt sick from all the agitation
In the past few weeks, it occurred that I suffered from pain in the stomach because of school
In the past few weeks, it occurred that I had strong headaches during class
*Social Problems In School (SPS)*
In the past few weeks, it occurred that I had problems with my classmates
In the past few weeks, it occurred that I had problems with single classmates
In the past few weeks, it occurred that I felt like an outsider in my classroom

Responses were indicated on a 6-point Likert scale ranging from 1 = *never/disagree* to 6 = *very often/agree*

**Table 5:** School Engagement Scale

*Behavioral Engagement (ENGB)*
I follow the rules at school
I get in trouble at school. (reversed)
When I am in class, I just act as if I am working. (reversed)
I pay attention in class
I complete my work on time
*Cognitive Engagement (ENGC)*
I check my schoolwork for mistakes
I study at home even when I don't have a test
I try to watch TV shows about things we do in school
When I read a book, I ask myself questions to make sure I understand what it is about
I read extra books to learn more about things we do in school
If I don't know what a word means when I am reading, I do something to figure it out
If I don't understand what I read, I go back and read it over again
I talk with people outside of school about what I am learning in class
*Emotional Engagement (ENGE)*
I like being at school
I feel excited by my work at school
My classroom is a fun place to be
I am interested in the work at school
I feel happy in school
I feel bored in school. (reversed)

Responses were indicated on a 5-point Likert scale ranging from 1 = *never* to 5 = *all of the time*

## Appendix 2

**Table 6:** Model 1: Enjoyment in School

Outcome	Predictor	Estimate	*SE*	95% CI
*Direct Effects*				
ENGB_t2_	EIS_t1_	0.613***	0.092	[0.432, 0.787]
	GPA_t1_	0.114	0.079	[-0.051, 0.25]
	ESCS	-0.011	0.051	[-0.117, 0.082]
ENGC_t2_	EIS_t1_	0.6***	0.075	[0.432, 0.723]
	GPA_t1_	-0.101	0.058	[-0.215, 0.012]
	ESCS	0.034	0.053	[-0.07, 0.137]
ENGE_t2_	EIS_t1_	0.764***	0.090	[0.601, 0.943]
	GPA_t1_	-0.017	0.077	[-0.17, 0.127]
	ESCS	0.042	0.057	[-0.072, 0.151]
GPA_t2_	EIS_t1_	-0.171	0.126	[-0.472, -0.01]
	ENGB_t2_	0.129*	0.054	[0.027, 0.242]
	ENGC_t2_	0.005	0.060	[-0.119, 0.117]
	ENGE_t2_	0.124	0.091	[-0.003, 0.319]
	GPA_t1_	0.77***	0.048	[0.67, 0.849]
	ESCS	0.08	0.042	[0.002, 0.165]
*Total Effect*				
	EIS_t1_ → GPA_t2_	0.006	0.047	[-0.084, 0.103]
*Indirect Effects*				
	EIS_t1_ → ENGB_t2_ → GPA_t2_	0.079*	0.037	[0.020, 0.173]
	EIS_t1_ → ENGC_t2_ → GPA_t2_	0.003	0.037	[-0.067, 0.076]
	EIS_t1_ → ENGE_t2_ → GPA_t2_	0.095	0.086	[0.002, 0.305]

EIS = enjoyment in school; ENGB = behavioral engagement, ENGC = cognitive engagement, ENGE = emotional engagement, ESCS = Socioeconomic Status; GPA = Grade Point Average; 95% CI = 95% bias-corrected bootstrap confidence interval; *t*1 = Wave 1; *t*2 = Wave 2

χ2 (260) = 1100.052, *p* < 0.001, RMSEA = 0.061, SRMR = 0.076, CFI = 0.823

**p* < 0.05, ***p* < 0.01, ****p* < 0.001

**Table 7:** Model 2: Positive Attitudes Toward School

Outcome	Predictor	Estimate	*SE*	95% CI
*Direct Effects*				
ENGB_t2_	PAS_t1_	0.516***	0.076	[0.364, 0.661]
	GPA_t1_	0.151**	0.056	[0.047, 0.266]
	ESCS	-0.002	0.048	[-0.103, 0.087]
ENGC_t2_	PAS_t1_	0.489***	0.060	[0.37, 0.604]
	GPA_t1_	-0.061	0.047	[-0.149, 0.037]
	ESCS	0.041	0.049	[-0.056, 0.139]
ENGE_t2_	PAS_t1_	0.713***	0.055	[0.61, 0.821]
	GPA_t1_	0.01	0.048	[-0.082, 0.107]
	ESCS	0.057	0.048	[-0.044, 0.147]
GPA_t2_	PAS_t1_	-0.052	0.077	[-0.218, 0.079]
	ENGB_t2_	0.102*	0.052	[0.001, 0.207]
	ENGC_t2_	-0.023	0.054	[-0.137, 0.077]
	ENGE_t2_	0.061	0.062	[-0.051, 0.19]
	GPA_t1_	0.757***	0.040	[0.665, 0.822]
	ESCS	0.082*	0.041	[0.006, 0.165]
*Total Effect*				
	PAS_t1_ → GPA_t2_	0.033	0.041	[-0.043, 0.120]
*Indirect Effects*				
	PAS_t1_ → ENGB_t2_ → GPA_t2_	0.053	0.029	[0.004, 0.121]
	PAS_t1_ → ENGC_t2_ → GPA_t2_	-0.011	0.027	[-0.066, 0.040]
	PAS_t1_ → ENGE_t2_ → GPA_t2_	0.043	0.048	[-0.035, 0.148]

PAS = positive attitudes toward school; ENGB = behavioral engagement, ENGC = cognitive engagement, ENGE = emotional engagement, ESCS = Socioeconomic Status; GPA = Grade Point Average; 95% CI = 95% bias-corrected bootstrap confidence interval; *t*1 = Wave 1; *t*2 = Wave 2

χ2 (260) = 1140.971, *p* < 0.001, RMSEA = 0.062, SRMR = 0.087, CFI = 0.827

**p* < 0.05, ***p* < 0.01, ****p* < 0.001

**Table 8:** Model 3: Positive Academic Self-concept

Outcome	Predictor	Estimate	*SE*	95% CI
*Direct Effects*				
ENGB_t2_	PASC_t1_	0.553**	0.192	[0.371, 1.065]
	GPA_t1_	0.052	0.163	[-0.338, 0.223]
	ESCS	-0.075	0.065	[-0.227, 0.02]
ENGC_t2_	PASC_t1_	0.438*	0.180	[0.26, 0.937]
	GPA_t1_	-0.114	0.132	[-0.412, 0.055]
	ESCS	-0.021	0.069	[-0.159, 0.105]
ENGE_t2_	PASC_t1_	0.523*	0.205	[0.337, 1.098]
	GPA_t1_	-0.02	0.163	[-0.423, 0.155]
	ESCS	-0.021	0.071	[-0.165, 0.095]
GPA_t2_	PASC_t1_	-0.044	0.108	[-0.276, 0.129]
	ENGB_t2_	0.107	0.066	[-0.012, 0.251]
	ENGC_t2_	-0.025	0.055	[-0.142, 0.074]
	ENGE_t2_	0.041	0.059	[-0.06, 0.17]
	GPA_t1_	0.764***	0.052	[0.66, 0.844]
	ESCS	0.09	0.050	[0.001, 0.196]
*Total Effect*				
	PASC_t1_ → GPA_t2_	0.025	0.050	[-0.059, 0.128]
*Indirect Effects*				
	PASC_t1_ → ENGB_t2_ → GPA_t2_	0.059	0.056	[-0.008, 0.210]
	PASC_t1_ → ENGC_t2_ → GPA_t2_	-0.011	0.032	[-0.092, 0.038]
	PASC_t1_ → ENGE_t2_ → GPA_t2_	0.021	0.050	[-0.031, 0.167]

PASC = positive academic self-concept; ENGB = behavioral engagement, ENGC = cognitive engagement, ENGE = emotional engagement, ESCS = Socioeconomic Status; GPA = Grade Point Average; 95% CI = 95% bias-corrected bootstrap confidence interval; *t*1 = Wave 1; *t*2 = Wave 2

χ2 (260) = 1232.026, *p* < 0.001, RMSEA = 0.065, SRMR = 0.106, CFI = 0.807

**p* < 0.05, ***p* < 0.01, ****p* < 0.001

**Table 9:** Model 4: Worries in School

Outcome	Predictor	Estimate	*SE*	95% CI
*Direct Effects*				
ENGB_t2_	WIS_t1_	-0.059	0.086	[-0.238, 0.102]
	GPA_t1_	0.295***	0.058	[0.178, 0.408]
	ESCS	-0.031	0.057	[-0.15, 0.075]
ENGC_t2_	WIS_t1_	0.108	0.084	[-0.057, 0.273]
	GPA_t1_	0.114	0.064	[-0.009, 0.243]
	ESCS	0.03	0.062	[-0.091, 0.152]
ENGE_t2_	WIS_t1_	-0.095	0.086	[-0.257, 0.078]
	GPA_t1_	0.206**	0.067	[0.075, 0.337]
	ESCS	0.019	0.066	[-0.108, 0.151]
GPA_t2_	WIS_t1_	0.072	0.037	[-0.001, 0.147]
	ENGB_t2_	0.09	0.049	[-0.005, 0.189]
	ENGC_t2_	-0.048	0.050	[-0.158, 0.041]
	ENGE_t2_	0.04	0.042	[-0.04, 0.123]
	GPA_t1_	0.766***	0.038	[0.68, 0.831]
	ESCS	0.092*	0.042	[0.009, 0.172]
*Total Effect*				
	WIS_t1_ → GPA_t2_	0.057	0.037	[-0.015, 0.129]
*Indirect Effects*				
	WIS_t1_ → ENGB_t2_ → GPA_t2_	-0.005	0.010	[-0.039, 0.006]
	WIS_t1_ → ENGC_t2_ → GPA_t2_	-0.005	0.009	[-0.038, 0.003]
	WIS_t1_ → ENGE_t2_ → GPA_t2_	-0.004	0.006	[-0.025, 0.003]

WIS = worries in school; ENGB = behavioral engagement, ENGC = cognitive engagement, ENGE = emotional engagement, ESCS = Socioeconomic Status; GPA = Grade Point Average; 95% CI = 95% bias-corrected bootstrap confidence interval; *t*1 = Wave 1; *t*2 = Wave 2

χ2 (260) = 1320.743, *p* < 0.001, RMSEA = 0.068, SRMR = 0.137, CFI = 0.793

**p* < 0.05, ***p* < 0.01, ****p* < 0.001

**Table 10:** Model 5: Social Problems in School

Outcome	Predictor	Estimate	*SE*	95% CI
*Direct Effects*				
ENGB_t2_	SPS_t1_	-0.201**	0.065	[-0.33, -0.075]
	GPA_t1_	0.289***	0.056	[0.174, 0.397]
	ESCS	-0.037	0.055	[-0.15, 0.066]
ENGC_t2_	SPS_t1_	-0.019	0.073	[-0.171, 0.112]
	GPA_t1_	0.087	0.062	[-0.028, 0.215]
	ESCS	0.017	0.064	[-0.105, 0.146]
ENGE_t2_	SPS_t1_	-0.237***	0.067	[-0.37, -0.111]
	GPA_t1_	0.203**	0.065	[0.074, 0.329]
	ESCS	0.013	0.065	[-0.113, 0.142]
GPA_t2_	SPS_t1_	0.01	0.026	[-0.041, 0.059]
	ENGB_t2_	0.09	0.047	[-0.003, 0.185]
	ENGC_t2_	-0.034	0.049	[-0.136, 0.056]
	ENGE_t2_	0.032	0.046	[-0.062, 0.12]
	GPA_t1_	0.753***	0.039	[0.664, 0.819]
	ESCS	0.086*	0.041	[0.005, 0.166]
*Total Effect*				
	SPS_t1_ → GPA_t2_	-0.015	0.025	[-0.068, 0.030]
*Indirect Effects*				
	SPS_t1_ → ENGB_t2_ → GPA_t2_	-0.018	0.013	[-0.051, 0.000]
	SPS_t1_ → ENGC_t2_ → GPA_t2_	0.001	0.004	[-0.005, 0.017]
	SPS_t1_ → ENGE_t2_ → GPA_t2_	-0.008	0.011	[-0.029, 0.016]

SPS = social problems in school; ENGB = behavioral engagement, ENGC = cognitive engagement, ENGE = emotional engagement, ESCS = Socioeconomic Status; GPA = Grade Point Average; 95% CI = 95% bias-corrected bootstrap confidence interval; *t*1 = Wave 1; *t*2 = Wave 2

χ2 (260) = 1277.625, *p* < 0.001, RMSEA = 0.067, SRMR = 0.134, CFI = 0.792

**p* < 0.05, ***p* < 0.01, ****p* < 0.001

**Table 11:** Model 6: Physical Complaints in School

Outcome	Predictor	Estimate	*SE*	95% CI
*Direct Effects*				
ENGB_t2_	PCS_t1_	-0.103	0.081	[-0.267, 0.044]
	GPA_t1_	0.288***	0.060	[0.164, 0.402]
	ESCS	-0.043	0.060	[-0.169, 0.068]
ENGC_t2_	PCS_t1_	0.164*	0.064	[0.03, 0.281]
	GPA_t1_	0.122	0.062	[0.001, 0.245]
	ESCS	0.048	0.065	[-0.08, 0.175]
ENGE_t2_	PCS_t1_	-0.145	0.080	[-0.309, 0.005]
	GPA_t1_	0.199**	0.070	[0.06, 0.334]
	ESCS	0.003	0.067	[-0.125, 0.138]
GPA_t2_	PCS_t1_	0.056	0.037	[-0.017, 0.128]
	ENGB_t2_	0.092	0.048	[-0.001, 0.19]
	ENGC_t2_	-0.049	0.051	[-0.161, 0.044]
	ENGE_t2_	0.04	0.046	[-0.053, 0.129]
	GPA_t1_	0.76***	0.037	[0.677, 0.824]
	ESCS	0.096*	0.044	[0.012, 0.183]
*Total Effect*				
	PCS_t1_ → GPA_t2_	0.033	0.033	[-0.036, 0.094
*Indirect Effects*				
	PCS_t1_ → ENGB_t2_ → GPA_t2_	-0.009	0.011	[-0.045, 0.002]
	PCS_t1_ → ENGC_t2_ → GPA_t2_	-0.008	0.010	[-0.038, 0.005]
	PCS_t1_ → ENGE_t2_ → GPA_t2_	-0.006	0.008	[-0.029, 0.004]

PCS = physical complaints in school; ENGB = behavioral engagement, ENGC = cognitive engagement, ENGE = emotional engagement, ESCS = Socioeconomic Status; GPA = Grade Point Average; 95% CI = 95% bias-corrected bootstrap confidence interval; *t*1 = Wave 1; *t*2 = Wave 2

χ2 (260) = 1358.838, *p* < 0.001, RMSEA = 0.066, SRMR = 0.135, CFI = 0.801

**p* < 0.05, ***p* < 0.01, ****p* < 0.001
